# Drug Consumption Rooms and Public Health Policy: Perspectives of Scottish Strategic Decision-Makers

**DOI:** 10.3390/ijerph19116575

**Published:** 2022-05-27

**Authors:** James Nicholls, Wulf Livingston, Andy Perkins, Beth Cairns, Rebecca Foster, Kirsten M. A. Trayner, Harry R. Sumnall, Tracey Price, Paul Cairney, Josh Dumbrell, Tessa Parkes

**Affiliations:** 1 Faculty of Health Sciences and Sport, University of Stirling, Stirling FK9 4LA, UK; 2 Faculty of Social and Life Sciences, Glyndwr University, Wrexham LL11 2AW, UK; w.livingston@glyndwr.ac.uk; 3 Figure 8 Consultancy, Dundee DD4 0HU, UK; andyperkins@f8c.co.uk (A.P.); beth@f8c.co.uk (B.C.); 4 Faculty of Social Sciences, University of Stirling, Stirling FK9 4LA, UK; rebecca.foster@stir.ac.uk (R.F.); tracey.price@stir.ac.uk (T.P.); j.l.dumbrell@stir.ac.uk (J.D.); t.s.parkes@stir.ac.uk (T.P.); 5 School of Health and Life Sciences, Glasgow Caledonian University, Glasgow G4 0BA, UK; kirsten.trayner@gcu.ac.uk; 6 Faculty of Health, Liverpool John Moores University, Liverpool L2 2QB, UK; h.sumnall@ljmu.ac.uk; 7 Faculty of Arts and Humanities, University of Stirling, Stirling FK9 4LA, UK; p.a.cairney@stir.ac.uk

**Keywords:** drug consumption rooms, safer injecting facilities, overdose prevention centres, drugs, policy, harm reduction, interventions, harm reduction, problem drug use, public health, Scotland

## Abstract

There is widespread support for the introduction of Drug Consumption Rooms (DCRs) in Scotland as part of a policy response to record levels of drug-related harm. However, existing legal barriers are made more complex by the division of relevant powers between the UK and Scottish Governments. This paper reports on a national, qualitative study of key decision-makers in both local and national roles across Scotland. It explores views on the political barriers and enablers to the adoption of Drug Consumption Rooms and the potential role of these facilities in the wider treatment system. It also considers approaches to evidence, especially the types of evidence that are considered valuable in supporting decision-making in this area. The study found that Scottish decision-makers are strongly supportive of DCR adoption; however, they remain unclear as to the legal and political mechanisms that would make this possible. They view DCRs as part of a complex treatment and support system rather than a uniquely transformative intervention. They see the case for introduction as sufficient, on the basis of need and available evidence, thus adopting a pragmatic and iterative approach to evidence, in contrast to an appeal to traditional evidence hierarchies more commonly adopted by the UK Government.

## 1. Introduction

Drug Consumption Rooms (DCRs; also sometimes referred to as Overdose Prevention Centres, Safe Injecting Facilities, or Safer Drug Consumption Facilities) are low-threshold settings for the supervised consumption of pre-obtained drugs, provision of clean injecting equipment, and intervention by trained staff in the event of an overdose [1,2]. Most DCRs also aim to support those not in contact with formal drug treatment through signposting to health and social care services and other advice such as welfare or benefits [1,3]. Over 100 DCRs currently operate in at least 66 cities in 10 countries, including sites in Europe, North America, and Australia [2,3,4,5]. International evidence suggests that DCRs may reduce the risk of blood-borne virus transmission, and thousands of on-site overdoses have been reversed [6,7]. However, study quality is limited, and research has only been conducted in a small number of sites [8].

Scotland is currently experiencing unprecedented levels of drug-related harm. Drug-related deaths are over three times the UK rate as a whole and among the highest in the world [9]. In 2020, Glasgow, Scotland’s largest city, had a death rate of 30.8 per 100,000 population and is also at the centre of an ongoing outbreak of HIV among people who inject drugs [9,10]. These high levels of harm and high rates of public injecting have led to proposals to introduce the UK’s first DCR in Glasgow [11,12]. DCRs have yet to be introduced in Scotland or elsewhere in the United Kingdom (UK), primarily because of legal barriers under the Misuse of Drugs Act 1971 (hereafter MDA). However, stakeholders in several local areas, and most notably in the devolved administration of Scotland, have called for DCRs to be opened. The UK Government has rejected these proposals, primarily on the grounds that they ‘encourage’ drug use, are a ‘distraction’ from other services, or simply remain illegal under current legislation [13,14,15]. 

While it has been argued that it would be possible to open a DCR if local police and other legal actors applied discretion not to prosecute [15,16,17,18], the operation of DCRs is associated with a range of potential offences, including under criminal and civil law. Therefore, specific legislation providing an exemption from criminal liability may be required [19]. DCRs represent a unique policy challenge within the constraints of a global drug policy system that prohibits the substances involved, although the International Narcotics Control Board has stated that DCRs are consistent with international drug control treaties so long as they form part of a wider treatment framework [19]. DCRs also illustrate the ‘jurisdictionally multi-level’ nature of drug policy [5], involving both local service providers and political authorities as well as regional or national decision-makers. 

Recent research has identified a range of challenges in establishing DCRs in areas where they have been proposed. These include insufficient political leadership [20,21]; community concerns over neighbourhood impacts [22,23]; public scepticism as to their value [24]; the impact of language and nomenclature [25,26]; and potential legal barriers [19]. The perceived policy goals of DCRs also vary. They can be seen as primarily solving the problems of amenity (e.g., discarded injecting equipment in public places, visible street drug use), public health (e.g., reducing overdose deaths or virus transmission), or marking a shift away from punitive approaches to drug problems and towards policies grounded in support and a reduction in stigma [2]. DCRs are, therefore, politically and conceptually complex in relation to wider systems of health and harm reduction.

### Drug Consumption Rooms in the Scottish Political Context

Public debate on DCRs in Scotland reflects a unique set of social and political conditions. High rates of drug harms have led to widespread calls for innovation, including the introduction of DCRs [11,27,28,29,30]. DCRs have also become the focus of advocacy among drug policy reformers (e.g., [31,32,33]). In 2020, the debate was intensified when an activist opened an unsanctioned (and, ostensibly, illegal) mobile Overdose Prevention Centre in Glasgow. The van operated without being closed by the police, and the high demand for the service added weight to advocacy for the introduction of DCRs [34]. The extensive media coverage that ensued created a ‘focusing event’ that amplified political pressure to introduce DCRs in response to Scotland’s drug death crisis [35,36,37].

Scotland enjoys significant devolved powers with respect to the rest of the United Kingdom, including health and justice policy [38]. Regarding drug treatment services, the Scottish Government determines expenditure and establishes the overarching strategy for the treatment system [39]. However, any amendments to the MDA are reserved for the UK Government (‘Westminster’). Under current UK law, providing a DCR could lead to charges under the MDA and other legislation [19]. In England and Wales, local police may exercise discretion in how they enforce the law and Police and Crime Commissioners can provide so-called ‘comfort letters’ allowing DCRs to operate [40]. In Scotland, the Lord Advocate (Scotland’s highest legal authority) has the authority to revise police standard operating procedures such that certain offences are not prosecuted and did so when expanding the use of Recorded Police Warnings for Class A drugs in 2021 [41]. In 2017, the then Lord Advocate declined to pursue such an approach, arguing that the provision of exemptions from criminal law was beyond the powers of his position [28]. However, in 2021 his successor said she would reconsider that decision [42]. 

Scotland has a recent history of health policy innovation. In 2018, the Scottish Government introduced a minimum unit pricing system for alcohol following a lengthy legal challenge from the alcohol industry, despite the Westminster Government rejecting the policy. Research has identified a number of reasons why Scotland innovated in this case, including significantly higher levels of alcohol harms in Scotland, a more coordinated advocacy coalition, closer ties between advocates, research communities, and Government, and a desire among Scottish politicians–especially those within the Scottish Nationalist Party–to pursue distinctive national health policy [43,44,45,46].

Scotland is, therefore, a distinctive policy ‘venue’ where unique political dynamics can inform decision-making [47]. Devolution makes policy innovation possible, but innovation can also become entangled in tensions between the Scottish and Westminster governments. This partial separation of responsibilities can also lead to uncertainty as to where the power sits when creating and implementing policy changes. In the case of drug policy, while the MDA is reserved for Westminster, the Scottish Government has been explicit in its desire to move towards a public health-oriented approach, in contradiction to a continuing emphasis on law enforcement-led responses at Westminster [39]. In 2019 the Scottish Drug Deaths Taskforce was established by the Minister for Public Health and Sport to better coordinate action on improving the health outcomes for people who use drugs. The taskforce has strongly advocated for public health-led drug policies, providing further evidence of this divergence [30]. These dynamics can shape both the way policy problems are defined and represented, and attitudes towards possible solutions. 

There is increasing research on public and political attitudes to DCRs, which seeks to better understand the barriers and enablers to their implementation in a range of contexts [19,20,21,22,23,24,25,26]. This paper explores the views and experiences of key strategic decision-makers who, were DCRs to be introduced in Scotland, would likely be involved in their implementation. Among the sample interviewed, there was universal support in principle for DCRs–albeit with a range of perspectives on their precise role and value. This paper, therefore, addresses two key policy questions:

(1) Why, given widespread support for DCRs, do strategic decision-makers feel adoption is proving so difficult?
(2) How do strategic decision-makers view DCRs as fitting into the wider policy and treatment systems?

It adds to the previous literature on the effectiveness of DCRs in reducing drug-related harm (e.g., [8]), and on public and political attitudes, by contributing a detailed, qualitative analysis of strategic decision-maker views in a context where DCRs have become highly politically charged.

## 2. Materials and Methods

These findings are drawn from a cross-sectional, qualitative study. An initial sampling framework was developed (AP, WL, TP, and RF), with the criteria that interviewees should be senior-level strategic decision-makers working in Scotland in roles that touch directly on current drug policy, harm reduction, or the prevention of drug-related deaths. The sample included Scottish Government officials, members of the Scottish Government Drug Death Taskforce, local Alcohol and Drug Partnerships (ADPs), local Health and Social Care Partnerships (HSCPs), local Integrated Joint Boards (IJBs), Community Justice, Community Safety, Police Scotland, third sector organisations, and national advocacy groups. 

AP, WL, RF, and TP2 developed a semi-structured topic guide with core themes, initial questions, and subsequent potential follow-ups, which was checked after four interviews for consistency of interviewer approach and data collection. The topic guide was further reviewed after 50% of the interviews were completed, and two additional questions were added about new government funding and the establishment of an unsanctioned mobile DCR in Glasgow. Overall, 38 individuals were approached, and 26 interviews were conducted. A comprehensive Scottish geographical spread was ensured, with interviewees being drawn from eight of 14 regional Health Board areas, as well as 11 interviewees who had a national remit. Some interviewees had more than one role of interest. The interviews were undertaken and recorded remotely by AP (n = 19) and WL (n = 7). Due to COVID-19 restrictions, interviews were carried out remotely on Teams and Zoom. The interviewees consented to quotation with full anonymity and are referred to below as DM (decision-maker), followed by the identification number allocated in the analysis.

Anonymised transcripts were analysed by BC in NVivo (v12) using a coding framework developed by AP. Two ‘theoretical triangulation’ [48] meetings were held to ensure consistency, and an analytical template was constructed with a priori themes. Initial codes were informed by these propositional starting points before additional emergent codes and sub-codes were added as the analysis progressed [49,50]. Throughout the process, regular meetings were held with the full research team to inform and refine the framework. The main study was supported by a Research Advisory Group including experts by experience. The topic guide was reviewed by this group, and minor changes were made in light of feedback. The group provided suggestions for the inclusion of certain stakeholders for interviews, which were passed on to the study team. Ethical approval was granted by the University of Stirling and Glyndwr University, Wrexham.

## 3. Results

There was consensus that drug-related deaths were a national crisis demanding novel political action. All of the interviewees supported the establishment or piloting of DCRs as part of this response. While the purpose of the study was in part to ascertain levels of support, in this paper we focus on four key themes that are pertinent in light of the fact that such support was expressed:

- interviewee perceptions of where decision-making power lay within the wider political system;
- how interviewees saw the role of DCRs in relation to the wider treatment system;
- approaches towards different roles, and types, of evidence in the debate;
- use of language in framing the purpose of, and principles behind, DCRs.

### 3.1. DCRs and Decision-Making in a Complex Political System

The interviewees were strongly aware of the tension between devolved powers and national legislation but also frustrated that DCRs had become a ‘political football’. A lack of clarity around the degree of autonomy held by Scottish authorities created not only a political blockage but also the opportunity for responsibility to be shifted in the face of a potentially contentious decision. Because the decision-making ‘venue’ was unclear, people could simultaneously support adoption while claiming responsibility for action lay elsewhere—thus shifting the imperative for action. 

For some, the primary problem was the lack of devolved powers to introduce DCRs and an unwillingness within Westminster for this to be addressed:

*It definitely shows the limitations of devolution, that we have something that is very clearly a public health issue, very clearly an area where it is the Scottish Government’s remit to try and fix this issue if it can be fixed. But there is that barrier there, which is a Westminster barrier—and not just with drug consumption rooms, just in terms of the Misuse of Drugs Act in its broadest sense and what it prevents you from doing*.

(DM12)

*It’s obviously Westminster. I’d like to think Scottish Government is actually genuine in its request for this rather than simply playing a card which they knew was going to get thrown out*.

(DM1)

For others, it was a lack of decisive action from the Scottish Government, alongside the tensions between Holyrood and Westminster, that was creating an impasse. The perception that a lack of national political decisiveness was preventing evidence-based action was a source of clear frustration:

*I would acknowledge it has at times been kicked around like a political football and when newspapers are looking for a news line, I can see why DCRs become the story. I think we have been caught between two Governments in this*.

(DM16)

*So perhaps the Government in Scotland need to be bold because this isn’t an argument about whether or not we devolve… I don’t know agriculture […] this is an argument about saving lives, and I think there is so much evidence out there that drug consumption rooms save lives*.

(DM14)

Inaction was also seen as a consequence of legislative uncertainty. The interviewees took a range of views on the role of the Lord Advocate in this, reflecting varied perceptions as to the precise powers held by the Lord Advocate. Some felt that the Lord Advocate could, and should, unilaterally provide police with assurances that they could allow DCRs to operate. Some pointed to a lack of explicit political support for such a move from Scottish Ministers, although, in reality, the decision-making powers of the Lord Advocate are fully independent of government. Others argued that the Lord Advocate’s power was legally constrained by UK law; though the provision of assurances would not, in fact, require a change to primary legislation:

*I think the Lord Advocate could be accused of bottling it by some […] as far as I can see needle exchange[s] are tolerated and I can’t see the difference*.

(DM1)

*[The Lord Advocate] will do exactly what the Government instruct him to do. So, if the Government really want a DCR, they would instruct him to issue a letter of comfort […] the Scottish Government could instruct him to do that tomorrow and he would find a way of doing that within the law*.

(DM5)

*[The Lord Advocate] doesn’t have the power to override UK law unless it’s in the public interest, and that’s only for prosecution, not for policing policy. So, there is a whole, it’s just another characteristic of the UK Government*.

(DM19)

The views expressed on the role of the Lord Advocate illustrate the degree to which, although high profile, and despite awareness that the Lord Advocate’s role was significant, there was a lack of clarity on precisely what their powers entailed. There was a broad sense that the Lord Advocate could play a decisive role, but mixed levels of understanding as to precisely how those powers–which, in reality, are independent, legal, and constitutional–operated in relation to the wider political system.

For some police respondents, the existing legal framework represented a specific problem, as they felt constrained to operate within the limits of laws which, without explicit guidance from the Lord Advocate, left little operational discretion:

*We are working within the law; we don’t have any say really in what the law is. So, we are working within the law and within public expectation. And we don’t have the discretion to just generally ignore a crime if it’s reported to us or happening in front of us*.

(DM7)

Prior studies have identified that while explicit community support is not essential to the establishment of a DCR, community concerns over nuisance or ‘honey pot’ effects can act as a key barrier to implementation [22]. Despite legislation permitting the establishment of DCR in Ireland (the Misuse of Drugs (Supervised Injecting Facilities) Act 2017), no facility has yet opened, in part due to community and business concerns over proposed locations. Interviewees saw community support as a critical factor in moving forward:

*We do need to have local community councils involved, as well as the elected members […] and the local authority actually needs to be involved […] I think we need to have as many local bodies as possible involved in that opening gambit, now that could make it an absolute quagmire and we might never get out of it. But we do need to bring together as many people as possible to start to move this forward*.

(DM4)

Overall, they presented a picture of a complex system in which no single stakeholder was seen as holding power to break the deadlock, either legally or politically. Political stasis was not viewed as due to determined opposition but rather perceived as the result of dispersed decision-making and an environment in which the capacity to move forward always sat elsewhere in the political system. 

### 3.2. The Role and Position of DCRs in the Wider Treatment System

The interviewees saw DCRs as part of a wide and complex system of public health promotion, treatment provision, and social support. They were not viewed as an intervention that stood outside of or could achieve benefits independently of the wider ecosystem of interventions. In this respect, there was a widespread rejection of the claim, often expressed by the Westminster Government, that DCRs were seen by supporters as a ‘silver bullet’ in regard to drug-related harms [51,52,53,54].

*And, of course, people who are not keen on DCRs will say “Well, it’s not the silver bullet!”. Well, no one is claiming that any one measure is the magic cure here. There is no magic cure*.

(DM16)

*It’s not one silver bullet because we are talking about quite a diverse group of drug users. They are not all the same […] They are not all affected in the same way and they don’t all have the same needs*.

(DM5)

*We are aware that it’s one of the things that is being promoted as the golden bullet to solve Scotland’s appalling drugs death problems. Personally, I’m not convinced. There is never a golden bullet on these issues. It’s a lot more complicated than one intervention, I think*.

(DM15)

The focus of most discussion was less the broad question of *whether* DCRs could reduce harms but where they should sit within wider support systems and whether implementation was politically feasible. Thus, while DCRs are often considered in isolation, with arguments focusing on their specific outcomes or the narrow ethical case for provision, interviewees applied a systemic frame to the problem: looking at structural contexts, their relationship to other interventions, and the impact of adoption on wider service sectors.

Many saw DCRs as working as an integrated service within and alongside other services:

*[W]e need a safe drug consumption facility … as part of the kind of range of services and interventions that we have in place for people with multiple and complex needs. So, I am absolutely clear on that*.

(DM10)

*So is there any reason, and I’m just thinking this off the top of my head, why we couldn’t integrate a kind of drug consumption space, or whatever way you want to phrase it, alongside our integrated homelessness services? Could we not do it, you know, alongside an integrated criminal justice service?*

(DM11)

*Our crisis centre in [city name removed] is a great example of this, where people come in for one thing and end up walking out with a lot more. So, they come in for a bit of advice or to get their gear, and while they are there they get blood spot tested and they get an HIV test, and they get whatever else and they get whatever else and a few leaflets to take away to say ‘Look if you are in crisis, for Pete’s sake, phone us’*.

(DM6)

Interviewees also identified possible limitations, especially around the practical reality of drug consumption rooms for potential clients. DCRs in fixed locations would be unavoidably limited in regard to geographical reach, and there were doubts as to how far people would be willing to travel to use the facilities. Similarly, respondents recognised that a clinical environment would not be attractive to all potential service users:

*When people are really in a bad place and they want to use, they are not going to go and buy their drugs, keep in in their pocket, then get on a bus […]. People that I know, who have taken drugs for a long time, want to do that in the comfort of their own home*.

(DM2)

DCRs were seen by a number of interviewees as shifting the focus of interventions away from a criminal justice-led response to drug harms. In this regard, DCRs represented not only an additional tool in the service system but also marked a value shift towards *‘saying that the person has a right to be treated with respect and dignity’* (DM5) and ensuring that opportunities to access further support were provided. In this way, DCRs were seen as achieving more than the narrow (though vital) goals of preventing overdoses and reducing viral transmission. Rather, they were viewed as an opportunity to create non-stigmatising environments that could provide opportunities to develop rapport and help guide people towards wider support. They also stood for a wider set of values which challenged the criminal justice-led response enshrined in both the existing legislation and public positioning of the Westminster Government. 

However, there was also concern about opportunity costs and the possible impact of the initial costs of implementing a DCR on the wider treatment system budget. This was seen as both a concrete financial problem and a political challenge around perceived priority-setting. An intensive political focus on DCRs was viewed by some interviewees as potentially leading to unrealistic expectations about what they might achieve or as diverting attention from other key parts of the treatment system.

*If the only investment, the only conversation we are having, is about drug consumption rooms then that is the wrong conversation. And we need to know what it costs, what is the financial envelope in relation to this?*

(DM1)

*So, I think the risk with something like drug consumption rooms is that government will give money expecting us to spend it on that, because that’s their preferred solution to the problem. And that might not be the best use*.

(DM15)

*The evidence suggests that, yes, there is a need for it. However, that should not be to the detriment of other parts of the system*.

(DM2)

Among the study participants, DCRs were not supported because they would entirely transform the system but because they represented an important contribution to provision. The heightened political attention they received was not necessarily welcome; the preferred outcome was for DCRs to become a standard treatment intervention, not a political totem or ongoing focus of debate.

### 3.3. Approaches to Evidence

The interviewees recognised that political viability required strong evidence that DCRs worked. However, their approach to evidence differed from that observed by Caulkins et al. (2019), who found that those involved in planning or allocating resources for DCRs would be more reliant on traditional evidence hierarchies (that is, clear results provided by randomised control trials and/or systematic reviews) compared to advocates or politicians [55]. Rather, decision-makers in this study tended to adopt what Cairney and Oliver (2017) have called an ‘improvement science’ approach: acknowledging that evidence development needed to be local, iterative, and coproduced while accepting that the weight of available international evidence was sufficient to justify at least pilot adoption [56]. At the same time, they felt that a key role of research evidence was to convince others in the policy system:

*Politically the evidence would need to be incredibly strong […] if […] there had to be a political and [Integrated Joint Board] decision*.

(DM3)

*There needs to be a fairly robust sort of evidence base around the use of drug consumption rooms. So, whether that’s through pilots or through studies from other countries or, but also include people with living or lived experience of drug use. I think as well around public perceptions*.

(DM17)

Our interviewees were less likely to be ‘sticklers wedded to traditional hierarchies of evidence’ [55] and more pragmatically concerned with how DCRs could be adopted within existing systemic constraints. Indeed, in this instance, it is the Westminster Government that has been most insistent on appealing to traditional evidence hierarchies—often citing ‘mixed evidence to show their effects’—in opposing DCR implementation [52]. For respondents in this study, the barrier was not traditional evidence of effectiveness but perceived roadblocks in the decision-making system that prevented innovation and local pilots.

### 3.4. The Role of Language in Framing DCRs

The interviewees understood that research evidence alone was not sufficient to support action, especially because DCRs involved a conceptual and practical break with previous norms [53]. There was a strong sense that the harm reduction principles underpinning DCRs had to be widely accepted, so progress was an issue of values, language, and framing. 

Nomenclature was seen as critical. Partly this was about values (‘drug consumption room’ potentially implying normalisation of drug use, while ‘overdose prevention centre’ focused on harm reduction), but also about perceptions as to what outcomes should be expected from the provision of DCRs (e.g., reduce overdoses, prevent nuisance or litter, provide a gateway to support etc.). It was also important to ensure that potential clients understood the function.

*‘Consumption’ I think is okay, ‘injecting’ is probably not because we would want to have a service […] which would not just be about injecting because lots of people use drugs in other ways. They may for example smoke heroin instead of injecting […] so I feel [safer injecting facilities] is a bit too narrow a term, plus it’s a bit in your face really as well.*

(DM14)

*The language does matter I think, so that people who might benefit from the service understand what it is and that it’s there. So, if you dress it up in opaque language then the people you are trying to reach won’t think this is for them. So, I think you know ‘drug consumption’ makes it obvious what it is. But I think that the language of safer injecting probably does take some of the fear factor away from you know, from residents, from other people who might feel a bit unsure*.

(DM16)

It was widely acknowledged that political action requires media support. In Scotland, there has been considerable media interest in DCRs, with several publications (including some that were traditionally viewed as conservative and opposed to drug policy reform) actively advocating for their introduction.

*The Herald and the Scotsman had editorials to support us, so eventually it only became the Daily Mail. Even the Evening Times changed their […] mind, which was fascinating. And colleagues in England were asking us about that because that was one of the things that they really struggled with and we had, we had national newspapers who had a different headline up here*.

(DM10)

However, the language used to describe attempts to introduce DCRs in the Scottish media varied and, even in news reports that were broadly supportive, could veer into cliché or stigmatising terms such as ‘fix rooms’ or ‘shooting galleries’ [20]. One interviewee starkly illustrated the relationship between language and stigma in this context:

*I still remember it was a comment, and I shouldn’t have read it, it was a comment to one of the earlier Evening Times articles and it was about yeah something, shooting galleries, except you should shut the door and shoot them all. I mean, I still remember that*.

(DM10)

Language and stigma also mattered in relation to pragmatic challenges in establishing wider community support. Decision-makers recognised that opposition existed within communities directly affected by drug problems. While this was a potential barrier to adoption, participants argued that better framing could effectively address those concerns:

*We talk about being inclusive and, you know, taking a health approach. Actually, for people living in a tenement where there is a drug user that has a dealer and their mates traipsing up and down the tenements, they just want rid of it.*

(DM15)

*In a place where you are trying to get people to address the stigma, even the words drug consumption rooms don’t sit particularly well with communities, with the cutting of council services all over the place, because the councils don’t have enough money for services*

(DM1)

Prior research reveals the impact of problem framing in shaping opinions on DCRs. Barry and colleagues (2019) show that negative views towards DCRs were underpinned by negative moral assessments of people who inject drugs (PWID) and that members of the public tended to place the greatest significance on the costs of provision rather than the role of DCRs in promoting dignity and respect towards PWID [24]. The decision-makers in this study recognised that policy change on DCRs needed evidence of effectiveness, reassurance of local communities, and non-stigmatising approaches to PWID, reflecting recent findings in regard to public opinion on DCRs by Sumnall and colleagues [26].

## 4. Discussion

There was a widely-held view among respondents that the potential public health gains to be achieved by introducing DCRs in Scotland were being held back by an unhelpful political stalemate. Ritter (2021: 84) has argued that because the policymaking context for decision-making around DCRs operates at multiple levels, they are particularly at risk of not being implemented due to a failure of the various policy ‘streams’ to align ([5,37]; see also [21] for the application of this concept to DCRs). Specifically, even when there is general agreement that drug deaths are a pressing social issue, the dispersion of decision-making responsibility makes it more difficult for the policy solution represented by DCRs to align with the political, ideological, or institutional contexts that apply. The more complex the decision-making system, the harder it is for one solution to be amenable to the array of political actors within that system. Smith et al. (2019), describing the successful introduction of a DCR in Liège, Belgium, adopt a similar model but focus on the relationships between intrinsic issue characteristics, political context, and the power of both political actors and ideas [57]. In Scotland, there has been a strong alignment of many of these factors: there is consensus that drug deaths are a crisis; the issue has become very high profile (albeit controversial), and national politicians—including Government—have acknowledged the need for innovation. The high level of support for DCRs among the decision-makers we interviewed appeared to reflect this. 

However, despite the widespread support for DCRs expressed during interviews, the fact that no formal DCR had been established at the time of the research suggests that political support had not been sufficiently energetic to motivate action in the face of political complexity. Interviewees communicated a clear view that DCRs formed only part of a wider treatment package, though the expressed rejection of a ‘silver bullet’ narrative shows how that framing had become prominent in the political debate even while it was not a view taken by any supporters. Some respondents remained uncertain, however, as to expenditure in relation to other solutions and were keenly aware of political risks. Therefore, the action came at higher possible costs, both political and opportunity, than retaining the status quo. As Unlu et al. (2022) found in Finland, arguments for the implementation of DCRs, although compelling, often failed by themselves to ‘constitute substantial leverage for policy change’, where high-level political leadership was missing [21]. However, as Smith et al. (2019) found in Belgium, the careful alignment of problem definition, policy framing, and stakeholder engagement can overcome such barriers [57].

While Westminster routinely appealed to traditional evidence hierarchies in rejecting DCRs, there was clear support for an ‘improvement science’ approach to DCRs among decision-makers: accepting the urgent need for innovation and assuming iterative development over time [56]. This pragmatic, small-scale approach is not uncommon in the Scottish political context [58]. It also provides a clear rationale for adoption since existing evidence is, by necessity, specific to local areas (primarily in Canada and Australia) where DCRs have been implemented and, in some cases, evaluated (e.g., [59,60]). However, for this approach to be adopted, decision-makers needed a clear steer from either the Scottish Government or the Lord Advocate. Individual stakeholders were unlikely to ‘go it alone’ without the confidence provided by high-level support.

In this study, the multi-level context for decision-making policymaking proved unhelpful in overcoming the obstacle of political opposition at Westminster. Although the Scottish Government has a history of health policy innovation, as was the case for minimum unit pricing for alcohol, it has so far not accepted the implementation of DCRs, despite openly supporting them in principle. Local policymakers could experiment, especially in light of the fact that local police did not close down the unauthorized Overdose Prevention Centre that opened in Glasgow in 2019, but they have so far remained unwilling [34]. This demonstrates that, while multi-level decision-making environments can be an opportunity for experimentation, they can equally present multiple opportunities for vetoes and inaction. The ‘vetoes’ need not represent active opposition but simply reluctance, a lack of resources, or a lack of sufficient motivation in the context of complex politics and competing claims on time and resources. Therefore, the problem is, arguably, not opposition but path dependency and an inertia characteristic of complex systems when dealing with controversial issues (see [20] for a further discussion in relation to DCRs).

The interviewees were less concerned with traditional evidence hierarchies than political pragmatism within a complex system or, in simpler terms, who had the power and willingness to give the ‘green light’ nationally and locally? How should local concerns be addressed? What would be the impact on wider systems? Our study found that it was not a matter of ‘better’ and ‘worse’ evidence but what types of evidence decision-makers viewed as sufficient to justify the introduction of a given intervention, especially in the context of a health crisis [55].

## 5. Limitations

The sampling for this study was purposive, and not all of those contacted agreed to be interviewed. Therefore, it may over-represent the perspectives of decision-makers with a particular interest in, stronger views on, or support for, drug consumption rooms. The sample was also limited to senior staff working at the intersection of strategy and policy, while the full stakeholder group for this topic is much broader. The findings of this study should be considered alongside evidence on the views of people who use drugs, treatment and support services, families, carers, and other affected groups. The research was carried out while COVID-19-related restrictions on social distancing were in place, necessitating online interviews rather than face-to-face. However, we felt this provided a more engaged interaction than a telephone interview while adding further flexibility in regard to participant availability. Overall, we do not feel the use of online platforms impacted negatively on the interview quality.

## 6. Conclusions

Our data show that rigid positions on different types of evidence were less central to the views of decision-makers than pragmatic political considerations. Furthermore, levels of support were determined by both values (e.g., belief in reducing stigma) and data on effectiveness. Respondents were overwhelmingly supportive of DCRs and confident that the available evidence justified their adoption. They were, however, keenly aware that these services formed part of a wider treatment and support system and were concerned with how DCRs would impact the wider structure. Rather than demanding more external evidence, interviewees were keen to adopt a flexible, iterative approach which engaged with local partners and stakeholders and provided PWID with the most attractive options in order to develop the best fit for local needs. The primary barrier to this was political, rather than evidential, with clear national leadership needed to empower local decision-makers to take the policy forward.

## Data Availability

Not applicable.

## References

[1] EMCDDA (2018). Perspectives on Drugs: Drug Consumption Rooms—An Overview of Provision and Evidence. https://www.emcdda.europa.eu/system/files/publications/2734/POD_Drug%20consumption%20rooms.pdf.

[2] Scottish Government (2021). Safer Drug Consumption Facilities—Evidence Paper. https://www.gov.scot/publications/safer-drug-consumption-facilites-evidence-paper/documents/.

[3] Schatz E., Nougier M. (2021). IDPC Briefing Paper: Drug Consumption Rooms Evidence and Practice. http://www.drugsandalcohol.ie/17898/1/IDPC-Briefing-Paper_Drug-consumption-rooms.pdf.

[4] EMCDDA (2018). Drug Consumption Rooms: An Overview of Provision and Evidence (Perspectives on Drugs). https://www.emcdda.europa.eu/publications/pods/drug-consumption-rooms_en.

[5] Ritter A. (2021). Drug Policy.

[6] Kennedy M.C., Karamouzian M., Kerr T. (2017). Public health and public order outcomes associated with supervised drug consumption facilities: A systematic review. Curr. HIV/AIDS Rep..

[7] Pardo B., Caulkins J.P., Kilmer B. (2018). Assessing the Evidence on Supervised Drug Consumption Sites. RAND Corporation Working Paper WR-1261-RC. https://www.rand.org/pubs/working_papers/WR1261.html.

[8] Kilmer B., Tayler J., Caulkins J.P., Mueller P.A., Allison A.J., Pardo B., Smart R., Strang L., Reuter P.H. (2018). Considering Heroin-assisted Treatment and Supervised Drug Consumption Sites in the United States.

[9] National Records of Scotland (2021). Drug-Related Deaths in Scotland in 2020. https://www.nrscotland.gov.uk/files/statistics/drug-related-deaths/20/drug-related-deaths-20-pub.pdf.

[10] McAuley A., Palmateer N.E., Goldberg D.J., Trayner K.M., Shepherd S.J., Gunson R.N., Metcalfe R., Milosevic C., Taylor A., Munro A. (2019). Re-emergence of HIV related to injecting drug use despite a comprehensive harm reduction environment: A cross-sectional analysis. Lancet HIV.

[11] Tweed E.J., Rogers M., Priyadarshi S., Crighton E. (2018). “Taking away the chaos”: A health needs assessment for people who inject drugs in public places in Glasgow, Scotland. BMC Public Health.

[12] Trayner K.M., McAuley A., Palmateer N.E., Goldberg D.J., Shepherd S.J., Gunson R.N., Tweed E.J., Priyadarshi S., Milosevic C., Hutchinson S.J. (2020). Increased risk of HIV and other drug-related harms associated with injecting in public places: National bio-behavioural survey of people who inject drugs. Int. J. Drug Policy.

[13] Anon Drug Consumption Rooms Are a ‘Distraction’ Says UK Minister. BBC News Online 27 February 2020. https://www.bbc.co.uk/news/uk-scotland-51644786.

[14] Anon Boris Johnson ‘Not in Favour’ of Drug Consumption Rooms. BBC News Online 4 August 2021. https://www.bbc.co.uk/news/uk-scotland-scotland-politics-58092735.

[15] House of Commons (2021). HC Deb 6 December—Ten Year Drug Strategy. https://hansard.parliament.uk/Commons/2021-12-06/debates/54065168-8B45-47B1-BEEB-1BA4B49DB4AF/Ten-YearDrugsStrategy.

[16] Easton M. Are UK Drug Consumption Rooms Likely? BBC News Online 12 October 2017. https://www.bbc.co.uk/news/uk-41596222.

[17] Clark J., Torrance J. (2018). Drug Consumption Room: Could the Provision of a Drug Consumption Room Bring Significant Benefit in Reducing Drug Related Deaths and Other Community Harms in Bristol—Feasibility Study. Bristol City Council. https://transformdrugs.org/assets/files/PDFs/Bristol-Supervised-Drug-Consumption-Facilities-feasibility-study-2.pdf.

[18] Tweed E., Rogers M. (2017). “Taking Away the Chaos”: The Health Needs of People Who Inject Drugs in Public Places in Glasgow City Centre. NHS Greater Glasgow and Clyde. https://www.nhsggc.org.uk/media/238302/nhsggc_health_needs_drug_injectors_full.pdf.

[19] Fortson R. (2017). Setting up a Drug Consumption Room—Legal Issues. Queen Mary School of Law Legal Studies Research Paper, No. 262/2017. https://papers.ssrn.com/sol3/papers.cfm?abstract_id=3061235.

[20] Unlu A., Demiroz F., Tammi T., Hakkarainen P. (2021). The complexity of drug consumption room policy and progress in Finland. Contemp. Drug Probl..

[21] Unlu A., Tammi T., Hakkarainen P. (2022). Policy windows for drug consumption rooms in Finland. Nord. Stud. Alcohol Drugs.

[22] Kolla G., Strike C., Watson T.M., Jairam J., Fischer B., Bayoumi A.A. (2017). Risk creating and risk reducing: Community perception of supervised consumption facilities for illicit drug use. Health Risk Soc..

[23] Taylor J., Ober A.J., Kilmer B., Caulkins J.P., Iguchi M.Y. (2021). Community perspectives on supervised consumption sites: Insights from four US counties deeply affected by opioids. J. Subst. Abus. Treat..

[24] Barry C.L., Sherman S.G., Stone E., Kennedy-Hendricks A., Niederdeppe J., Linden S., McGinty E.E. (2019). Argument supporting and opposing legalization of safe consumption sites in the US. Int. J. Drug Policy.

[25] Socia K.M., Stone R., Palacios W.R., Cluverius J. (2021). Focus on prevention: The public is more supportive of “overdose prevention sites” than they are of “safe injection facilities”. Criminol. Public Policy.

[26] Sumnall H.R., Atkinson A.M., Trayner K.M.A., Gage S.H., McAuley A. (2020). Effects of messaging on public support for drug consumption rooms in Scotland. Int. J. Drug Policy.

[27] Atkinson A.M., McAuley A., Trayner K.M.A., Sumnall H.R. (2019). ‘We are still obsessed buy this idea of abstinence’: A critical analysis of UK news media representations of proposals to introduce drug consumption rooms in Glasgow, UK. Int. J. Drug Policy.

[28] House of Commons Scottish Affairs Committee (2019). Problem Drug Use in Scotland—First Report of Session 2019. House of Commons HC44. https://www.parliament.uk/business/committees/committees-a-z/commons-select/scottish-affairs-committee/inquiries/parliament-2017/problem-drug-use-scotland-17-19/.

[29] House of Commons (2021). HC Deb 17 June 2021—Misuse of Drugs Act. https://hansard.parliament.uk/Commons/2021-06-17/debates/A1B14B26-EBB7-415F-9AA8-1620726307C5/MisuseOfDrugsAct.

[30] Scottish Drug Deaths Task Force (2021). Report on Drug Law Reform. https://drugdeathstaskforce.scot/media/1248/drug-law-reform-report-sept-6th-21.pdf.

[31] Drug Science (2022). Enhanced Harm Reduction Working Group. https://www.drugscience.org.uk/enhanced-harm-reduction-working-group/.

[32] Scottish Drugs Forum (2022). Drug Consumption Facilities—What You Need to Know. https://www.sdf.org.uk/drug-consumption-facilities-need-know/.

[33] Transform Drug Policy Foundation (2022). A Proven Way to Save Lives. https://transformdrugs.org/drug-policy/uk-drug-policy/overdose-prevention-centres.

[34] Shorter G.W., Harris M., McAuley A., Trayner K.M.A., Stevens S. (2022). The UK’s first (unsanctioned) safe injecting facility: Observations of the first six months. Int. J. Drug Policy.

[35] Alimi E.Y., Maney G.M. (2018). Focusing on Focusing Events: Event Selection, Media Coverage, and the Dynamics of Contentious Meaning-Making. Soc. Forum.

[36] Krykant P. Why I Set up the UKs First Drug Consumption van. Guardian. 30 April 2021. https://www.theguardian.com/commentisfree/2021/apr/30/uk-first-drug-consumption-van.

[37] Kingdon J. (2010). Agendas, Alternatives and Public Policies.

[38] Scottish Parliament Devolved and Reserved Powers—What Can the Scottish Parliament Decide?. https://www.parliament.scot/about/how-parliament-works/devolved-and-reserved-powers.

[39] Scottish Government (2018). Rights, Respect and Recovery: Alcohol and Drug Treatment Strategy. https://www.gov.scot/publications/rights-respect-recovery/documents/.

[40] Eastwood N. (2019). Drug-related deaths: Playing politics while people die. BMJ Opin..

[41] Crown Office and Procurator Fiscal Service (2021). Lord Advocate Statement on Diversion from Prosecution. https://www.copfs.gov.uk/media-site-news-from-copfs/1983-lord-advocate-statement-on-diversion-from-prosecution.

[42] Hutcheon P. (2021). Lord Advocate suggests fresh look at drug consumption rooms in Scotland. Daily Record.

[43] Katikireddi S.V., Hilton S., Bonell C., Bond L. (2014). Understanding the development of minimum unit pricing in Scotland: A qualitative study of the policy process. PLoS ONE.

[44] Katikireddi S.V., Hilton S., Bond L. (2014). The role of the Sheffield model on the minimum unit pricing alcohol debate: The importance of a rhetorical perspective. Evid. Policy.

[45] Patterson C., Katikireddi S.V., Wood K., Hilton S. (2014). Representations of minimum unit pricing for alcohol in UK newspapers: A case study of a public health policy debate. J. Public Health.

[46] Butler S., Elmeland K., Nicholls J., Thom B. (2017). Alcohol, Power and Public Health.

[47] Cairney P. (2007). Using devolution to set the agenda?: Venue shift and the smoking ban in Scotland. Br. J. Politics Int. Relat..

[48] Denzin N.K. (1970). The Research Act in Sociology.

[49] Corbin J., Strauss A. (1990). Grounded Theory research: Procedures, canons and evaluative criteria. Qual. Sociol..

[50] Braun V., Clarke V. (2006). Using thematic analysis in psychology. Qual. Res. Psychol..

[51] Rodger H., ‘No evidence’ to support safe consumption rooms, UK Government drugs adviser warns (2021). The Herald.

[52] Malthouse K. (2022). Scottish and UK governments must work together to reduce drugs deaths and tackle criminal gangs that profit from misery and death. The Scotsman.

[53] Bol D. (2021). Douglas Ross: Drug Consumption Rooms should not be ruled out. The Herald.

[54] Scottish Parliament (2022). Criminal Justice Committee, Health, Social Care and Sport Committee, and Social Justice and Social Security Committee (Joint Meeting)—Session 6: Reducing Drug Deaths in Scotland and Tackling Problem Drug Use. Col 12. https://www.parlamaid-alba.scot/api/sitecore/CustomMedia/OfficialReport?meetingId=13564.

[55] Caulkins J.P., Pardo B., Kilmer B. (2019). Supervised consumption sites: A nuanced assessment of the causal evidence. Addiction.

[56] Cairney P., Oliver K. (2017). Evidence-based policymaking is not like evidence-based medicine, so how far should you go to bridge the divide between evidence and policy?. Health Res. Policy Syst..

[57] Smith P., Favril L., Delhauteur D., Vander Laenen F., Nicaise P. (2019). How to overcome political and legal barriers to the implementation of a drug consumption room: An application of the policy agenda framework to the Belgian situation. Addict. Sci. Clin. Pract..

[58] Cairney P. (2017). Evidence-based practice is more political than it looks: A case study of the ‘Scottish Approach’. Evid. Policy.

[59] Kennedy M.C., Hayashi K., Milloy M.-J., Wood E., Kerr T. (2019). Supervised injection facility use and all-cause mortality among people who inject drugs in Vancouver, Canada: A cohort study. PLoS Med..

[60] Tran V., Reid S.E., Roxburgh A., Day C.A. (2021). Assessing drug consumption rooms and longer term (5 year) impacts on community and client. Risk Manag. Healthc. Policy.

